# Right-to-left shunt due to iatrogenic atrial septal defect manifested by aorto-caval fistula: a case report

**DOI:** 10.1186/s40981-024-00735-y

**Published:** 2024-08-15

**Authors:** Takuya Kimura, Takuya Okada, Norihiko Obata, Yasushi Motoyama, Masaharu Nagae

**Affiliations:** 1 Department of Anesthesiology and Pain Clinic, Hyogo Prefectural Harima-Himeji General Medical Center, 3-264 Kamiya-Cho, Himeji, Hyogo 670-8560 Japan; 2 Division of Anesthesiology, Department of Surgery Related, Kobe University Graduate School of Medicine, 7-5-2 Kusunoki-Cho, Chuo-Ku, Kobe, Hyogo 650-0017 Japan

**Keywords:** Aorto-caval fistula, Right-to-left shunt, Intra-aortic balloon occlusion, Iatrogenic atrial septal defect

## Abstract

**Background:**

An aorto-caval fistula is a rare but critical complication of abdominal aortic aneurysm (AAA) rupture, leading to high-output heart failure and increased venous pressure. The anesthetic management of such cases, particularly when complicated by an intraoperative right-to-left shunt, is seldom reported.

**Case presentation:**

A 71-year-old man with a history of atrial fibrillation and catheter ablation presented with heart failure and abdominal pain, leading to cardiac arrest. Imaging revealed an AAA rupture into the inferior vena cava. During emergency surgery, severe venous bleeding was managed using intra-aortic balloon occlusion (IABO). Transesophageal echocardiography (TEE) identified a right-to-left shunt due to an iatrogenic atrial septal defect.

**Conclusion:**

Early TEE recognition and timely IABO intervention were crucial in managing this complex case, underscoring the importance of these techniques in similar emergency scenarios.

**Supplementary Information:**

The online version contains supplementary material available at 10.1186/s40981-024-00735-y.

## Background

An aorto-caval fistula (ACF) is a rare complication associated with an abdominal aortic aneurysm (AAA) rupture, occurring in 1–4% of cases with a perioperative mortality rate of 7–19% [1–4]. Urgent surgical intervention is recommended for AAA rupture leading to ACF. However, the rapid shunting through the fistula can cause high-output heart failure and increase venous pressure, posing challenges for anesthesia management.

Anesthesiologists play a crucial role in such cases, but specific anesthesia strategies are not well-documented.

In recent years, the frequency of catheter ablation procedures for patients with atrial fibrillation (AF) has been increasing [5]. Consequently, the number of cases requiring anesthetic management for these patients is also rising. Therefore, anesthesiologists must be aware of the precautions when anesthetizing these patients.

Here, we present the anesthesia management of a rare case with a right-to-left shunt identified intraoperatively using transesophageal echocardiography (TEE), likely due to an iatrogenic atrial septal defect (iASD) from previous catheter ablation therapy.

## Case presentation

**Fig. 1:**
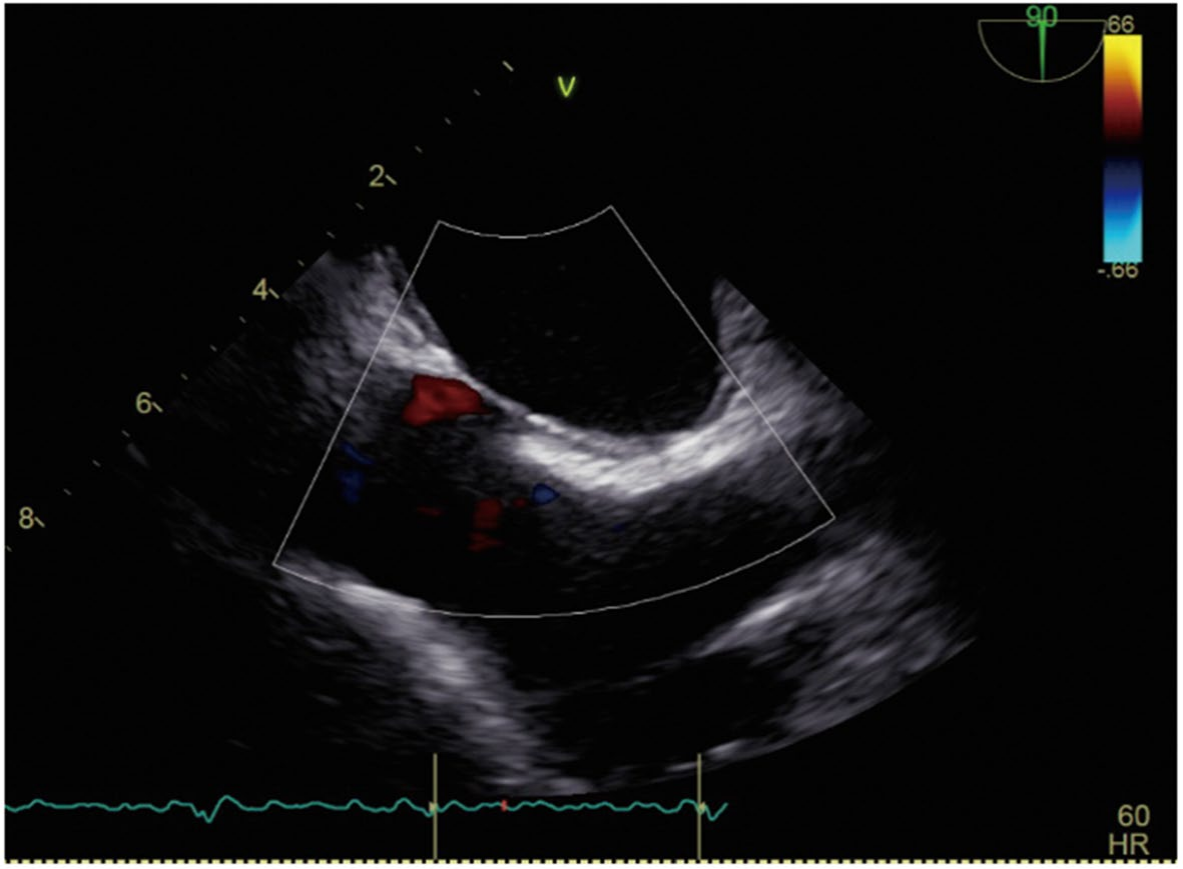
In the transesophageal echocardiogram taken within 3 months after catheter ablation treatment, there was no evidence of a patent foramen ovale or an iatrogenic ASD, nor were any other intracardiac shunts. The image shows the superior and inferior vena cava of the middle esophagus

A 71-year-old man underwent catheter ablation for AF 4 years earlier. During follow-up, there were no indications of a patent foramen ovale or an iASD, nor were any other intracardiac shunts detected during subsequent TEE (Fig. 1). He was hospitalized for recurrent AF and heart failure symptoms, including palpitations and fatigue. On admission, transthoracic echocardiography (TTE) showed a mildly reduced ejection fraction of 41%, without significant valvular abnormalities or intracardiac shunts. While receiving inpatient treatment for heart failure, the patient suddenly reported abdominal pain, which was followed by cardiac arrest. Immediate life-saving measures, including chest compressions, tracheal intubation, and administration of epinephrine, restored spontaneous circulation.

**Fig. 2:**
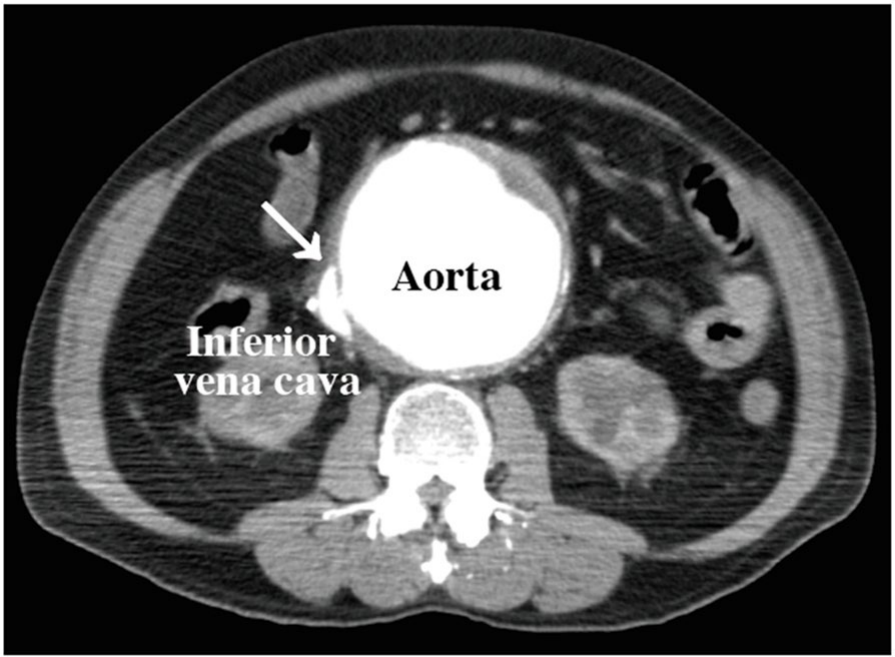
On contrast-enhanced computed tomography, an aorto-caval fistula that occurred due to the rupture of an abdominal aortic aneurysm was identified

Contrast-enhanced computed tomography (CT) of the chest and abdomen revealed a rupture of the AAA into the IVC (Fig. 2). No intracardiac shunts were identified on CT. Due to limited space near the renal arteries, endovascular stent graft placement was deemed inappropriate, necessitating emergency open abdominal aortic replacement surgery.

Before surgery, the patient had a blood pressure of 100/50 mmHg with continuous norepinephrine infusion at 0.2 μg/kg/min, a central venous pressure (CVP) of 35 mmHg, a pulmonary artery pressure (PAP) of 43/37 mmHg, an irregular pulse rate of 120 beats/min (AF), oxygen saturation of 97% (FiO_2_ 1.0), and a body temperature of 35.0 °C. The patient’s hemoglobin level was 13.2 g/dL upon admission but decreased to 10.6 g/dL at the start of surgery. On TTE, cardiac contractility had remained unchanged, but signs of increased right heart strain were observed.

General anesthesia was planned. The airway was secured through endotracheal intubation, and central venous access was established during resuscitation. Standard monitoring along with arterial blood pressure (ABP), CVP, PAP, electroencephalogram (BIS®), cerebral oximeter (INVOS®), and neuromuscular transmission (TOF-Watch®) was utilized. We monitored bladder temperature and maintained the body temperature using a warming device (Bair Hugger®). Cerebral oxygen saturation upon arrival in the operating room was approximately 50% bilaterally. Anesthetic induction was initiated using 1% sevoflurane, 100 μg of fentanyl, and 50 mg of rocuronium. Anesthesia was maintained using sevoflurane, rocuronium boluses, and fentanyl boluses. Immediately when surgery was started, substantial venous bleeding was observed from various sites, including the incision site. We used the Belmont® Rapid Infuser System to administer fluids and blood transfusions at a maximum rate of 500 mL/min for managing large-volume fluid needs.

**Fig. 3:**
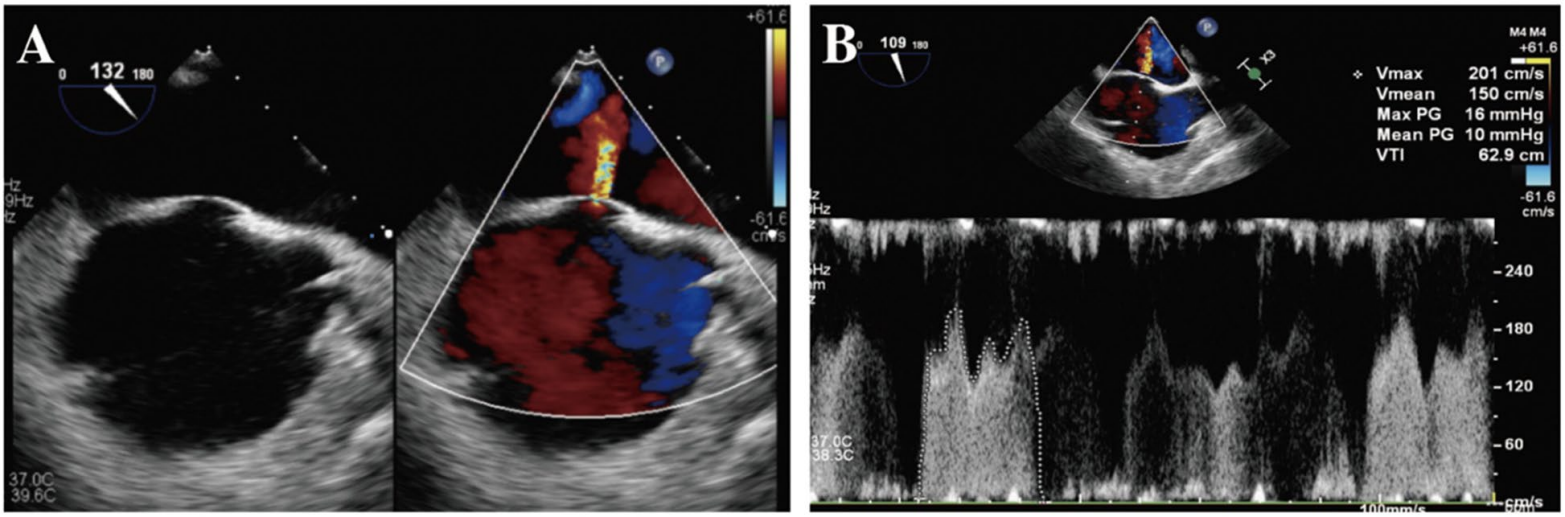
**A** Intraoperative transesophageal echocardiography showed a right-to-left shunt through the foramen ovale. **B** The peak pressure gradient was 16 mmHg

**Fig. 4:**
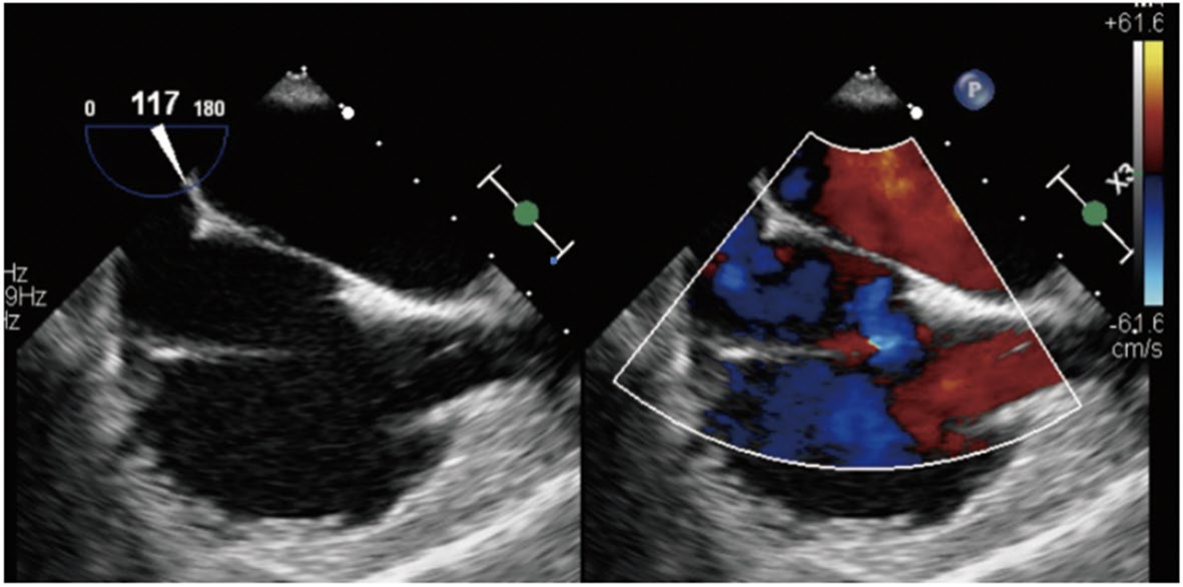
After intra-aortic balloon occlusion (IABO), the right-to-left shunt at the central part of the foramen ovale was no longer detectable

**Fig. 5:**
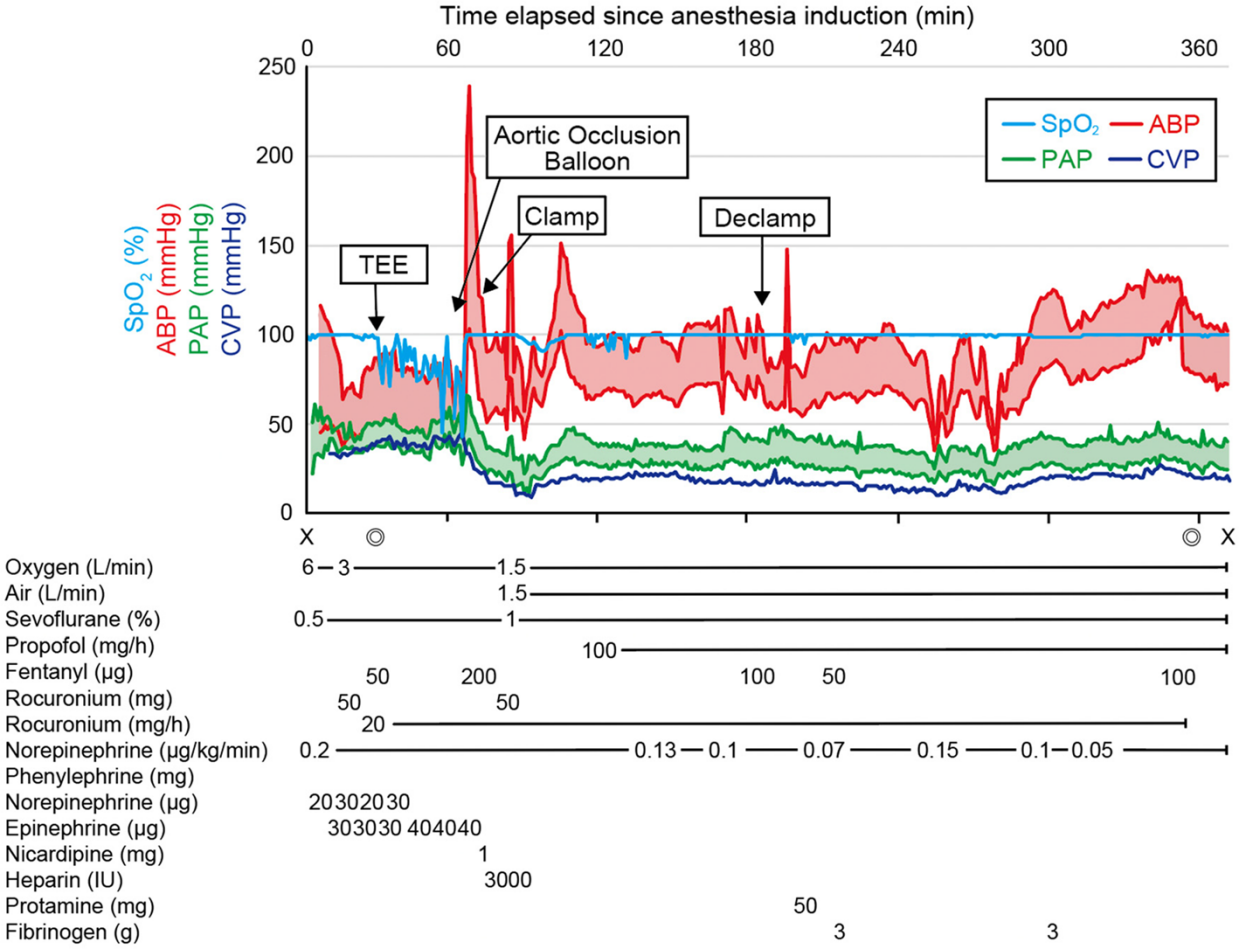
Anesthesia record. TEE, transesophageal echocardiography; X, start and end of anesthesia; ◎start and end of surgery; SpO_2_, saturation of percutaneous oxygen; ABP, arterial blood pressure; PAP, pulmonary artery pressure; CVP, central venous pressure

Despite massive blood transfusions, continuous norepinephrine infusion at 0.2 μg /kg/min, and repeated bolus epinephrine of 30–40 μg, ABP decreased to 50/20 mmHg with persistent increase of CVP to 35 mmHg. The hemoglobin level reached its lowest point of 7.2 g/dL. Cerebral oxygen saturation decreased to 20%. TEE revealed a right-to-left shunt through the central foramen ovale with a peak pressure gradient of 16 mmHg, while both left and right ventricular systolic function remained unchanged (Fig. 3, Supplementary Video). An intra-aortic balloon occlusion (IABO) was inserted in the descending thoracic aorta from the femoral artery and immediately resulted in an increase of ABP to 140/50 mmHg with a decreased of CVP to 10 mmHg and PAP to 25/10 mmHg. Cerebral oxygen saturation increased to 50–60%. TEE revealed disappearance of right-to-left shunt (Fig. 4), and venous bleeding was controlled. However, arterial bleeding persisted, and fibrinogen level was 89 mg/dL, necessitating transfusion of fresh-frozen plasma and fibrinogen concentrate of 6 g before successful surgical hemostasis. The surgery proceeded well, closing the fistula with a patch, and replacing the abdominal aorta with a Y-shaped artificial graft. The surgery lasted 339 min with an estimated blood loss which was 21,557 mL. Transfusions included 3360 mL of packed red blood cells, 7200 mL of fresh-frozen plasma, 600 mL of platelet concentrate, and 8120 mL of autologous salvaged blood (Fig. 5). At the end of the surgery, the hemoglobin level had increased to 10.5 g/dL, and fibrinogen levels had risen to 210 mg/dL. Due to significant bleeding and massive transfusion, the body temperature dropped to 34.0 °C. In the ICU, the patient underwent warming, resulting in a return to approximately 36.0 °C within 4 h. Bilateral pleural effusions and significant edema were identified postoperatively, taking time to resolve. Extubation was performed on the 6th postoperative day, and the patient was discharged home without other complications on day 30.

## Discussion

AAA rupture into the IVC can present with various clinical signs, including abdominal pain, palpable aneurysm, distension of abdominal wall veins, hematuria, and signs of heart failure such as pulmonary congestion and increased venous pressure [6, 7]. Given the nature of the disease, shunting through the fistula can lead to high-output heart failure, especially right-sided, and the rapid shunting can lead to fatal venous bleeding due to increased venous pressure. Accurate early diagnosis and prompt emergency surgery are crucial [8]. There are very few reports of the anesthetic management in emergency surgery for AAA rupture into the IVC [9, 10]. There are two important considerations for intraoperative management during these cases.

One critical consideration is the timing of IABO placement. In cases of AAA rupture leading to an ACF, it is vital for managing severe venous bleeding and hemodynamic instability. IABO is typically placed as soon as hemodynamic instability or significant venous bleeding is identified. The goal is to rapidly reduce venous pressure and control bleeding, thereby stabilizing the patient for definitive surgical repair [11]. Ideally, IABO should be placed in a controlled setting such as an operating room or interventional radiology suite before induction of anesthesia [12]. However, in emergency situations where rapid intervention is necessary, it may be placed in the emergency department. In some cases, IABO is placed during the initial stages of surgery once the diagnosis of ACF is confirmed. This helps to manage bleeding during the preparation for aneurysm repair and fistula closure.

Another important consideration is the possibility of developing intracardiac shunts in patients after ablation. Anesthesiologists should be aware that in these patients, even in the absence of previously documented intracardiac shunts, significant intracardiac shunting may develop depending on the hemodynamic conditions [13]. We experienced a rare case with a right-to-left shunt identified intraoperatively using TEE, likely due to an iASD from previous catheter ablation therapy. In surgeries for patients with significantly elevated hydrostatic pressure due to ACF, as in this case, it is essential to perform TEE early to detect the presence of intracardiac shunts.

In this case, after cardiac activity was restored through cardiopulmonary resuscitation, hemodynamic stability was maintained with a continuous norepinephrine infusion. Our initial focus was on arterial cross-clamping to reduce the blood flow in the ACF, which proved difficult due to substantial bleeding from the incision site and surrounding adipose tissue. We opted for IABO, which effectively controlled the bleeding without the need for other options such as partial IVC clamping, balloon catheter insertion in the IVC, or cardiopulmonary bypass [14, 15].

A possible factor in managing this case successfully could be the alleviation of right heart failure through a right-to-left shunt from the iASD. The patient had prior catheter ablation for AF, which included a percutaneous septal puncture. However, follow-up of TEE and TTE showed no remaining iASD. This case suggests that ACF development might have raised right atrial pressure, possibly resulting in the reappearance of a previously closed iASD. Typically, iASDs resolve in 96.3% of patients within 3 months [16]. However, emergency transcatheter closure procedures have been performed in cases of worsening oxygenation [17, 18]. In this case, the iASD may have alleviated right-sided pre-load, potentially preventing severe complications like multi-organ failure and cardiac arrest from worsening right heart failure. Moreover, the right-to-left shunt from the iASD could have caused complications like paradoxical embolism and hypoxemia. However, IABO placement reduced the shunt volume through the ACF, managing the condition without these complications.

In summary, TEE facilitated the early identification of a right-to-left shunt from the iASD during emergency surgery for ruptured AAA with ACF. Subsequent appropriate interventions were successful. A patient with a ruptured AAA accompanied by an ACF in whom a right-to-left shunt was identified due to an iASD was managed successfully using IABO. Ruptured AAA with ACF can lead to severe complications like right heart failure and venous hemorrhage, emphasizing the importance of early TEE recognition and prompt IABO intervention.

### Supplementary Information

Supplementary Material 1: Supplementary Video. Intraoperative transesophageal echocardiography showed a right-to-left shunt through the foramen ovale

## Data Availability

Not applicable.

## Abbreviations

ACF: Aorto-caval fistula
AAA: Abdominal aortic aneurysm
IVC: Inferior vena cava
AF: Atrial fibrillation
iASD: Iatrogenic atrial septal defect
IABO: Intra-aortic balloon occlusion
ABP: Arterial blood pressure
CVP: Central venous pressure
PAP: Pulmonary artery pressure
TEE: Transesophageal echocardiography
TTE: Transthoracic echocardiography
